# The Diagnosis and Initial Management of Children Presenting With Premature Loss of Primary Teeth Associated With a Systemic Condition

**DOI:** 10.7759/cureus.62402

**Published:** 2024-06-14

**Authors:** Hafiz Ali Shabbir Rajput, Akbar Ahmed, Afreen Bilgrami, Beenish Haider, Jamal Nasir Khan, Muhammad Afnan

**Affiliations:** 1 Department of Medicine, Liaquat National Hospital, Karachi, PAK; 2 Department of Gynaecology and Obstetrics, Ziauddin Medical University, Karachi, PAK; 3 Department of Dental Materials, Fatima Jinnah Dental College, Karachi, PAK; 4 Department of Dental Materials, Akhter Saeed Medical and Dental College, Lahore, PAK; 5 Department of Oral Biology, Gandhara University, Peshawar, PAK; 6 Cardiovascular Medicine, Khyber Medical Institute of Medical Sciences, Kohat, PAK

**Keywords:** initial management, diagnostic approaches, systemic conditions, premature tooth loss, pediatric dentistry

## Abstract

Background and objective

Pediatric dentists face a serious challenge when encountering cases of primary teeth lost too soon due to systemic disorders such as diabetes mellitus, congenital heart disease, and chronic kidney disease. Prompt identification and treatment are necessary to minimize problems in these patients. This study aimed to better understand and enhance clinical outcomes in pediatric dentistry treatment by investigating diagnostic modalities and early therapy methods for kids who lose their primary teeth too soon because of systemic disorders.

Methodology

We conducted a retrospective observational study to examine the early loss of primary teeth in children aged 6-10 years with a history of systemic diseases at Naseer Teaching & MMC-General Hospital, Peshawar; Hayatabad Medical Complex, Peshawar; DHQ Teaching Hospital, Kohat; and Fauji Foundation Hospital, Multan from January to December 2022. After carefully gathering data from medical records, a sample of 360 patients meeting the inclusion criteria was examined. SPSS Statistics version 27 (IBM Corp., Armonk, NY) was used for the statistical analysis. Demographic characteristics, clinical manifestations, and management approaches were compiled using descriptive statistics. For categorical data, frequency distributions and percentages were determined, and for continuous variables, means and standard deviations (SD) were calculated. Regression analysis was conducted to analyze relationships between related variables and treatment outcomes. A p-value <0.05 was considered statistically significant.

Results

The majority of patients were aged between six and eight years, and the cohort had an equal gender distribution. Dental problems including malocclusion (n=175, 48.61%) and early tooth loss (n=245, 68.06%) were common, as were systemic illnesses like genetic disorders (n=45, 12.50%) and endocrine abnormalities (n=67, 18.61%). Diagnostic procedures were often carried out, such as radiographic exams (n=256, 71.11%) and blood tests (n=123, 34.17%). Dietary supplements (n=60, 16.67%) and dental procedures (n=75, 20.83%) constituted the bulk of the treatment. Significant treatment outcomes that demonstrated the efficacy of the therapies were as follows: high patient satisfaction (n=213, 59.17%), improved oral health (n=255, 70.83%), and symptom relief (n=187, 51.94%).

Conclusion

Our findings highlight the significant impact of certain practical methods for identifying and treating early tooth loss in pediatric patients with systemic illnesses, leading to patient satisfaction in terms of symptom relief and enhanced dental health.

## Introduction

Early identification and treatment are crucial in pediatric dentistry to mitigate the impact of premature loss of primary teeth due to systemic diseases, thereby minimizing potential repercussions [1]. The early loss of primary teeth can result from various systemic illnesses, such as autoimmune diseases, dietary inadequacies, endocrine abnormalities, and genetic disorders [2,3]. These conditions may interfere with the primary teeth's regular growth and eruption, causing early exfoliation that might endanger the child's general health and oral hygiene [4,5]. For an accurate diagnosis and prompt care, it is important to understand the etiology and clinical symptoms of premature primary tooth loss in the context of systemic diseases [6]. The early diagnosis of underlying systemic disorders that contribute to tooth loss enables targeted treatment options addressing dental and systemic health issues holistically [7]. Effective treatment from the initial visit itself may also reduce the likelihood of later dental issues, including malocclusion, loss of arch length, and the psychological effects of premature tooth loss [8].

In routine clinical practice, pediatric dentists, pediatricians, and experts in related medical fields must collaborate to diagnose and treat children with early loss of primary teeth linked to systemic disorders [9]. Essential stages in the diagnosis process include obtaining a complete medical and dental history, a comprehensive clinical examination, and necessary diagnostic tests [10]. The differential diagnosis may include ruling out common causes of early tooth loss, such as dental caries and traumatic injuries while taking into account less frequent systemic illnesses presenting with comparable oral signs [11]. After a diagnosis, the main goal of treating these patients involves treating the underlying systemic ailment and any dental consequences that may arise [12]. The techniques to achieve optimum oral health and development may include anticipatory guidance, dental procedures to protect existing teeth and support appropriate occlusion, and managing systemic disease [13]. Early intervention may include working in tandem with other medical specialists to provide all-encompassing treatment tailored to meet the specific requirements of the child [14].

The purpose of this study was to better understand and optimize clinical outcomes in pediatric dentistry treatment by investigating diagnostic modalities and early therapy methods for kids who lose their primary teeth too soon because of systemic disorders.

## Materials and methods

Study design and settings

The retrospective observational research was conducted from January to December 2022. This study design was chosen due to its ability to efficiently utilize existing medical records to analyze historical data, making it ethically feasible and cost-effective while allowing for comprehensive statistical analysis to identify patterns and relationships between systemic diseases and early tooth loss in children. The study was conducted at several tertiary care hospitals, including Naseer Teaching & MMC-General Hospital, Peshawar; Hayatabad Medical Complex, Peshawar; DHQ Teaching Hospital, Kohat; and Fauji Foundation Hospital, Multan. These hospitals are equipped with specialized pediatric and dental departments, catering to a diverse array of patients with varying medical needs.

Inclusion and exclusion criteria

The inclusion criteria were as follows: children between the ages of six and 10 with early primary tooth loss due to systemic diseases including oral discomfort, early tooth loss, gingival irritation, malocclusion, oral infections, delayed eruption of permanent teeth, abnormalities of oral mucosa, endocrine problems, genetic diseases, dietary inadequacies, hematologic illnesses, autoimmune diseases, gastrointestinal disorders, neurological problems, and respiratory illnesses. Documentation of diagnostic testing, such as blood tests and radiographic exams, had to have been conducted along with early management initiatives like nutritional supplements or dental care. The participants were also required to produce their full medical records for review in the future. Patients aged below six years and above 10 years, and those with unavailable relevant medical records were excluded from the study.

Sample size

During the study period, medical records were used to identify 360 eligible patients who met the inclusion criteria. To guarantee sufficient representation and enough statistical power, the sample size was determined based on predicted prevalence rates and statistical considerations. The software used for sample size calculation was G*Power.

Data collection

The data collection process involved a thorough evaluation of the medical and dental records of the patients, including information about their demographics, medical histories, dental examination results, radiographic reports, and treatment plans. Systematic documentation was also done on related systemic diseases, including information on diagnostic procedures and treatment plans. To identify systemic problems associated with early tooth loss in children, specific criteria were employed during the data collection process. These criteria included documented diagnoses of endocrine problems, genetic diseases, dietary inadequacies, hematologic illnesses, autoimmune diseases, gastrointestinal disorders, neurological problems, and respiratory illnesses in the medical records of eligible participants.

Endocrine problems were defined as diagnoses such as hormonal imbalances or disorders affecting the endocrine system. Genetic diseases included conditions with a known genetic etiology or inheritance pattern. Dietary inadequacies were identified based on documented nutritional deficiencies or dietary habits contributing to poor oral health. Hematologic illnesses included blood disorders or abnormalities affecting hematopoiesis. Autoimmune diseases comprised conditions characterized by immune system dysfunction and autoantibody production. Gastrointestinal disorders encompassed conditions affecting the digestive tract or associated organs. Neurological problems included diagnoses related to the central or peripheral nervous system. Respiratory illnesses were defined as diagnoses like respiratory tract infections, chronic respiratory conditions, or pulmonary diseases.

These criteria were applied consistently to ensure accurate identification and documentation of systemic problems in the study population. Systemic conditions were diagnosed and confirmed through detailed medical records, including blood tests, imaging studies, and specific diagnostic evaluations. To reduce possible biases and standardize data gathering, structured data collecting forms were used. Accuracy and completeness were ensured by the methodical evaluation and extraction of pertinent data from medical records by skilled research staff.

Statistical analysis

SPSS Statistics version 27 (IBM Corp., Armonk, NY) was used for the statistical analysis. Demographic traits, clinical manifestations, and management approaches were compiled using descriptive statistics. For categorical data, frequency distributions and percentages were determined, and for continuous variables, means and standard deviations (SD) were calculated. Comparison studies, including regression analysis, were carried out where necessary to investigate relationships between related variables and treatment results. A p-value <0.05 was considered statistically significant.

Ethical approval

The study adhered to the ethical standards of the institutional research committees and the 1964 Helsinki Declaration and its later amendments or comparable ethical standards. Ethical approval was obtained from the Ethics Review Committees of Naseer Teaching and MMC-General Hospital, Peshawar. Given the retrospective nature of the study, the requirement for informed consent was waived by the committee; however, verbal informed consent was obtained from the guardians or relatives of the participants. Throughout the research process, stringent confidentiality measures were upheld to protect patient privacy.

## Results

The demographic features of the cohort are shown in Table 1. The mean age of the patients was 7.6 ± 0.9 years; 118 patients (32.78%) were aged six to seven years, 163 patients (45.28%) were aged seven to eight, 79 patients (21.94%) were aged eight to nine, and 40 patients (11.11%) were aged 9-10 years. The cohort comprised 184 female patients (51.11%) and 176 male patients (48.89%). In terms of socioeconomic status, 98 patients (27.22%) were classified as low, 183 patients (50.83%) as intermediate, and 79 patients (21.94%) as high. The educational attainment of parents varied; 93 (25.83%) had completed elementary school, 151 (41.94%) had completed secondary school, and 116 (32.22%) had a college or university degree. As for the distribution of family sizes, 132 patients (36.67%) were from families with fewer than four members, 156 (43.33%) were from families with four to six members, and 72 patients (20.00%) were from families with six or more people.

**Table 1:** Demographic features of the study cohort (n=360) SD: standard deviation

Variable	Category	Number of patients	Percentage
Age, years	6-7	118	32.78
	7-8	163	45.28
	8-9	79	21.94
	9-10	40	11.11
Mean age ± SD, years	7.6 ± 0.9		
Gender	Male	176	48.89
	Female	184	51.11
Parental socioeconomic status	Low	98	27.22
	Middle	183	50.83
	High	79	21.94
Parental education	Primary school	93	25.83
	Secondary school	151	41.94
	College/university	116	32.22
Family size	<4	132	36.67
	4-6	156	43.33
	>6	72	20.00

Table 2 provides a comprehensive analysis of various oral health-related factors among 360 participants. It delineates the prevalence of different conditions such as oral discomfort (n=85, 23.61%), early tooth loss (n=245, 68.06%), gingival irritation (n=132, 36.67%), malocclusion (n=175, 48.61%), oral infections (n=121, 33.61%), delayed eruption of permanent teeth (n=97, 26.94%), abnormalities of oral mucosa (n=53, 14.72%), endocrine problems (n=67, 18.61%), genetic diseases (n=45, 12.50%), dietary inadequacies (n=48, 13.33%), hematologic illnesses (n=19, 5.28%), autoimmune diseases (n=53, 14.72%), gastrointestinal disorders (n=38, 10.56%), neurological problems (n=24, 6.67%), and respiratory illnesses (n=66, 18.33%).

**Table 2:** Prevalence of oral health and early systemic disease-related variables among the participants (n=360)

Variable	Value	Number of participants	Percentage
Oral discomfort	Yes	85	23.61%
	No	275	76.39%
Early tooth loss	Yes	245	68.06%
	No	115	31.94%
Gingival irritation	Yes	132	36.67%
	No	228	63.33%
Malocclusion	Yes	175	48.61%
	No	185	51.39%
Oral infections	Yes	121	33.61%
	No	239	66.39%
Delayed eruption of permanent teeth	Yes	97	26.94%
	No	263	73.06%
Abnormalities of oral mucosa	Yes	53	14.72%
	No	307	85.28%
Endocrine problems	Yes	67	18.61%
	No	293	81.39%
Genetic diseases	Yes	45	12.50%
	No	315	87.50%
Dietary inadequacies	Yes	48	13.33%
	No	312	86.67%
Hematologic illnesses	Yes	19	5.28%
	No	341	94.72%
Autoimmune diseases	Yes	53	14.72%
	No	307	85.28%
Gastrointestinal disorders	Yes	38	10.56%
	No	322	89.44%
Neurological problems	Yes	24	6.67%
	No	336	93.33%
Respiratory illnesses	Yes	66	18.33%
	No	294	81.67%

**Figure 1:**
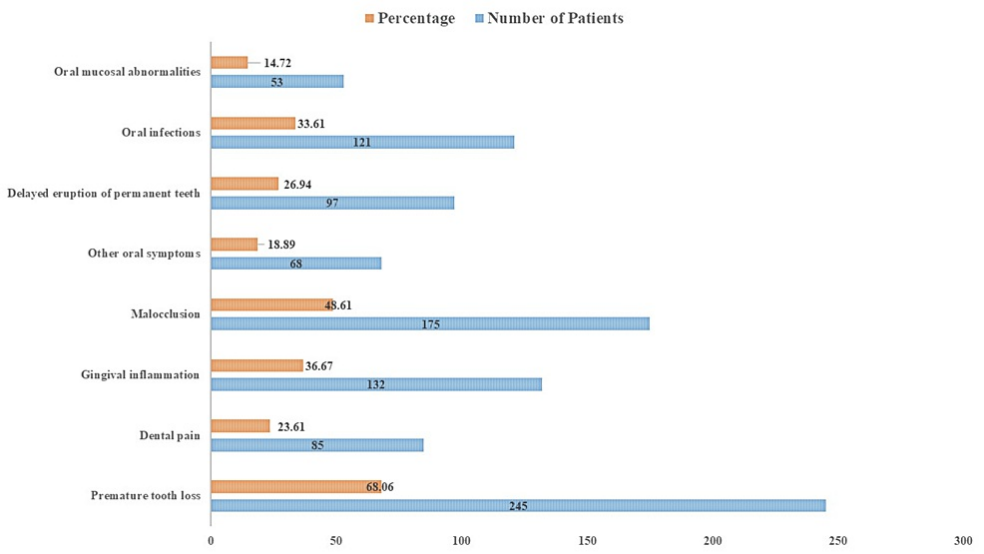
Clinical presentations among the cohort

The clinical manifestations among juvenile patients experiencing early loss of primary teeth are shown in Figure 1. Of the patients, 85 (23.61%) felt oral discomfort, while 245 (68.06%) had early tooth loss. Additionally, 132 (36.67%) had gingival irritation, 175 (48.61%) had malocclusion, and 68 (18.89%) had other oral symptoms. Furthermore, oral infections were found in 121 (33.61%) patients, and delayed eruption of permanent teeth afflicted 97 (26.94%) patients. Abnormalities of the oral mucosa were observed in 53 (14.72%) patients.

**Figure 2:**
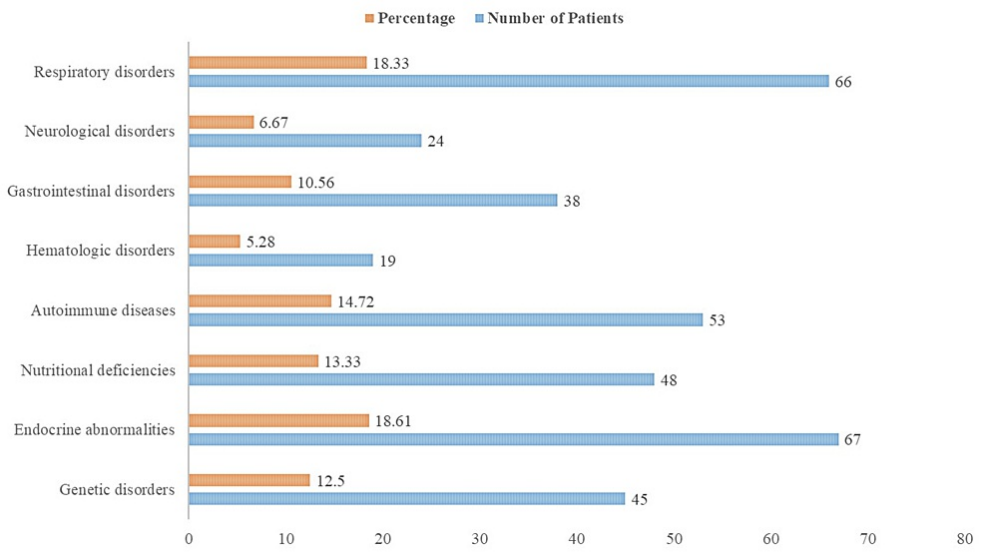
Associated systemic conditions in the cohort

Figure 2 sheds light on the systemic problems linked to children who lose their primary teeth too soon. Endocrine problems were seen in 67 patients (18.61%), genetic diseases in 45 patients (12.50%), and dietary inadequacies in 48 patients (13.33%). Hematologic illnesses affected 19 patients (5.28%), autoimmune diseases had 53 patients (14.72%), and gastrointestinal disorders affected 38 patients (10.56%). Furthermore, 24 patients (6.67%) had neurological problems, and 66 patients (18.33%) had respiratory illnesses.

The diagnostic procedures performed among the patients are listed in Table 3. Of note, 123 patients (34.17%) underwent blood testing, and 256 patients (71.11%) underwent radiographic exams. Endocrine evaluations were done on 145 patients (40.28%), and genetic testing on 89 patients (24.72%). A histopathological examination was performed for 54 patients (15.00%), while 76 patients (21.11%) underwent immunological testing. Microbiological testing was conducted for 98 patients (27.22%), and salivary analysis was done for 112 patients (31.11%).

**Table 3:** Diagnostic examinations performed among the cohort

Diagnostic test	Number of patients	Percentage
Blood tests	123	34.17
Radiographic examinations	256	71.11
Genetic testing	89	24.72
Endocrine assessments	145	40.28
Histopathological examination	54	15.00
Immunological tests	76	21.11
Microbiological tests	98	27.22
Salivary analysis	112	31.11

Treatment plans implemented for the patients are shown in Table 4. The majority of patients received dental treatments, with 75 patients (20.83%) choosing this option, highlighting the significance of oral health. Sixty patients (16.67%) received dietary supplements to address nutritional deficits contributing to tooth loss. Fifty patients underwent surgical procedures (13.89%), indicating the need for more intrusive therapies in certain circumstances. Dental procedures included extractions, restorations, and space maintainers to address dental issues and ensure proper alignment. Dietary supplements were used to correct nutritional deficiencies impacting dental health and support tissue repair. Additional therapies encompassed a comprehensive approach tailored to the specific needs of each patient, including medical management (n=45, 12.50%), orthodontic treatment (n=30, 8.33%), pharmacological therapy (n=40, 11.11%), psychological assistance (n=25, 6.94%), and speech therapy (n=35, 9.72%).

**Table 4:** Treatment modalities among the cohort

Treatment	Number of patients	Percentage
Medical management	45	12.50
Dental interventions	75	20.83
Orthodontic treatment	30	8.33
Dietary supplementation	60	16.67
Pharmacological therapy	40	11.11
Surgical interventions	50	13.89
Psychological support	25	6.94
Speech therapy	35	9.72

Table 5 lists the treatment results and related variables in the cohort. The majority of patients had excellent outcomes, as validated by the significant results: 187 patients (51.94%) had their symptoms resolved, 255 patients (70.83%) experienced an improvement in their oral health, and 142 patients (39.44%) saw their condition stabilize. Patients expressed high levels of satisfaction, with 213 patients (59.17%) stating that they were happy with the care they received. Additionally, 176 patients (48.89%) experienced an improvement in their quality of life. On the other hand, 98 patients (27.22%) required further therapy, while 43 patients (11.94%) experienced adverse consequences. The results showed statistical significance in the areas of symptom relief (p<0.001), oral health improvement (p<0.001), and patient satisfaction (p<0.001).

**Table 5:** Treatment results and associated factors in the cohort

Treatment outcome	Number of patients	Percentage	Coefficient (β)	Standard error	P-value
Resolution of symptoms	187	51.94	0.582	0.134	<0.001
Improvement in dental health	255	70.83	0.451	0.112	<0.001
Stabilization of the condition	142	39.44	0.312	0.098	0.003
Adverse effects	43	11.94	-0.201	0.076	0.009
Need for further treatment	98	27.22	-0.147	0.065	0.025
Patient satisfaction	213	59.17	0.387	0.105	<0.001
Quality of life improvement	176	48.89	0.264	0.094	0.005
Long-term prognosis	125	34.72	0.179	0.082	0.031

## Discussion

Our findings provide important fresh perspectives on the diagnosis and initial treatment approaches for children who lose their primary teeth too soon due to systemic disorders. Regarding demographic features, we observed that the cohort predominantly had an equal distribution in terms of age group and gender, with the vast majority of participants aged between six and years. In particular, 118 patients (32.78%) were aged six to seven years, 163 patients (45.28%) were aged seven to eight years, and 176 patients (48.89%) were male. These results underline the need for early interventions targeting this age group and are in line with previous research [15,16].

Our patients showed a significant incidence of malocclusion (48.61%) and early tooth loss (68.06%) in their clinical presentations. Other oral symptoms were also often noted, including gingival irritation (36.67%) and delayed eruption of permanent teeth (26.94%). Moreover, 33.61% of the patients had oral infections. Our findings regarding the frequency of clinical presentations are consistent with other investigations on juvenile patients who had early loss of primary teeth due to systemic diseases [4,5]. The complex nature of dental problems in children with systemic diseases is highlighted by these results, which warrants an all-encompassing approach to diagnosis and treatment.

Genetic disorders (12.50%) such as Down syndrome and cystic fibrosis, endocrine abnormalities (18.61%) like diabetes mellitus and hypothyroidism were the most common related systemic problems, followed by autoimmune diseases (14.72%) (juvenile idiopathic arthritis and systemic lupus erythematosus) and dietary deficiencies (13.33%) such as deficiencies in vitamins D and C, and calcium. Because endocrine problems are so common, further research is necessary to determine how they affect oral health outcomes. The study's results on the incidence of related systemic illnesses are consistent with earlier studies on pediatric patients who had early loss of primary teeth due to systemic disorders [17].

The diagnostic procedures carried out in this investigation, particularly the blood tests (34.17%) and radiographic exams (71.11%), are in line with accepted protocols for assessing dental and systemic health in young patients with early tooth loss linked to systemic diseases. These results are consistent with other studies [18,19], highlighting the critical roles played by radiography and blood analysis in determining the systemic causes causing early tooth loss. Our research does, however, highlight several deficiencies, such as the underutilization of histological examination (15.00%) and genetic testing (24.72%), which point to the need for better integration of these instruments in clinical practice. By addressing these gaps, future studies may investigate ways to improve diagnostic precision and prompt action.

Dental therapies (20.83%) and nutritional supplements (16.67%) such as those employed to address deficiencies in vitamins D and C, and calcium, constituted the majority of treatment regimens in this research, indicating a multimodal approach to controlling early tooth loss associated with systemic diseases. Consistent with other studies [20], our results highlight the significance of both curative and preventative approaches in pediatric dentistry. A small percentage of our patients (13.89%) required surgical procedures, highlighting the difficulty in treating this patient group and the occasional necessity for specialized therapies.

The efficacy of therapies in treating premature tooth loss in this group has been demonstrated by significant treatment results, such as high patient satisfaction (59.17%), improvement in oral health (70.83%), and relief of symptoms (51.94%). These encouraging results emphasize how crucial early diagnosis and interdisciplinary care are to maximizing clinical results and enhancing the quality of life for impacted youngsters. These results were further supported by comparison with earlier research [21], which emphasized the constancy of treatment effects across various patient demographics and healthcare settings.

Limitations

The study's focus on patients aged 6-10 years may not fully capture the spectrum of pediatric cases affected by early tooth loss. The sample size of 360 patients, while substantial, may not adequately represent the diverse population of children with systemic illnesses experiencing this dental issue. The use of statistical analyses, while helpful in exploring relationships between variables, may not fully account for confounding factors or other variables influencing treatment outcomes. The absence of long-term follow-up data limits the assessment of treatment efficacy over time. However, despite these limitations, the study contributes valuable insights into improving clinical outcomes in pediatric dentistry and underscores the importance of early identification and intervention in managing dental problems associated with systemic disorders.

Suggestions for future research

Future studies could explore more precise diagnostic tools, such as advanced imaging techniques and biomarker analysis, to better identify systemic conditions linked to early tooth loss. Integrating care models involving pediatricians, dentists, and nutritionists could enhance holistic treatment approaches. Long-term investigations are needed to assess treatment outcomes effectively. Innovative treatments and targeted nutritional interventions should be explored to improve patient outcomes. Additionally, research into the genetic and molecular mechanisms underlying systemic diseases and dental health could help devise personalized treatment strategies.

## Conclusions

We believe our findings contribute immensely to the proper diagnosis and treatment of early tooth loss in children with systemic disorders. A multidisciplinary approach and early intervention are essential for symptom improvement, oral health, and patient satisfaction. Extensive diagnostic assessments, including radiographic exams and blood testing, are essential for determining the systemic causes of tooth loss. Even though the majority of patients showed favorable outcomes, further investigation is required to improve diagnostic precision and explore cutting-edge treatments to achieve superior long-term results in this population.
